# Efficient and Quality-Optimized Metagenomic Pipeline Designed for Taxonomic Classification in Routine Microbiological Clinical Tests

**DOI:** 10.3390/microorganisms10040711

**Published:** 2022-03-25

**Authors:** Sylvie Buffet-Bataillon, Guillaume Rizk, Vincent Cattoir, Mohamed Sassi, Vincent Thibault, Jennifer Del Giudice, Jean-Pierre Gangneux

**Affiliations:** 1 Inserm, Institut NUMECAN (Nutrition Metabolisms and Cancer), CHU Rennes, Univ Rennes, F-35000 Rennes, France; sylvie.buffet-bataillon@chu-rennes.fr; 2 ILLUMINA, F-35000 Rennes, France; guillaume.rizk@gmail.com (G.R.); jennifer.delgiudice@gmail.com (J.D.G.); 3 Inserm, U1230 (ARN Régulateurs Bactériens et Médecine), CHU Rennes, Univ Rennes, F-35000 Rennes, France; vincent.cattoir@chu-rennes.fr (V.C.); mohamed.sassi@chu-rennes.fr (M.S.); 4 Inserm, EHESP, IRSET (Institut de Recherche en Santé, Environnement et Travail)—UMR_S 1085, CHU Rennes, Univ Rennes, F-35000 Rennes, France; vincent.thibault@chu-rennes.fr

**Keywords:** microbiome, mycobiome, virome, metagenomics, shotgun, Kraken2, Bracken, Kaiju, quality assessment, clinical microbiology

## Abstract

Metagenomics analysis is now routinely used for clinical diagnosis in several diseases, and we need confidence in interpreting metagenomics analysis of microbiota. Particularly from the side of clinical microbiology, we consider that it would be a major milestone to further advance microbiota studies with an innovative and significant approach consisting of processing steps and quality assessment for interpreting metagenomics data used for diagnosis. Here, we propose a methodology for taxon identification and abundance assessment of shotgun sequencing data of microbes that are well fitted for clinical setup. Processing steps of quality controls have been developed in order (i) to avoid low-quality reads and sequences, (ii) to optimize abundance thresholds and profiles, (iii) to combine classifiers and reference databases for best classification of species and abundance profiles for both prokaryotic and eukaryotic sequences, and (iv) to introduce external positive control. We find that the best strategy is to use a pipeline composed of a combination of different but complementary classifiers such as Kraken2/Bracken and Kaiju. Such improved quality assessment will have a major impact on the robustness of biological and clinical conclusions drawn from metagenomic studies.

## 1. Introduction

The role of the human microbiome in medicine has become of paramount importance, and this emerging field is rife with opportunities for discovery. The strategy of developing microbiome/mycobiome-based biomarkers for predicting disease risk is one of the most promising, particularly during systemic inflammatory diseases, metabolic diseases, and cancers [1]. Large international research programs and more than 10,000 scientific papers containing the keyword “microbiome” have been published each year for 5 years. However, approaches for high-throughput sequencing and analysis of microbiome from various origins (mainly respiratory, digestive, or urinary tracts and skin) results are increasingly diverse. First, the goals differ from pathophysiological studies to diagnosis, until the comparison of human and environmental microbiomes with different matrixes such as water, air, and surfaces [2]. Second, the methodology also greatly varies with different technologies (short versus long reads) and multiple sequencing equipment, different approaches (targeted versus shotgun metagenomics), or different targets (microbiome versus mycobiome versus virome [2]. Finally, bioinformatic analysis of millions of sequences is a key challenge, and the reproducibility of pipelines is essential. Ye and colleagues published a remarkable paper on the benchmarking of metagenomics tools for taxonomic classification [3]. Their study examined the performance of 20 metagenomic classifiers using datasets by comparing the size and growth of reference databases, along with specific key metrics: precision-recall of the classifiers, abundance profile distances for 20 classifiers, and the computational requirements. Among the DNA classifiers, Kraken and its derivate tools Bracken, KrakenUniq, and Kraken2 have several advantages. Indeed, they provide good performance metrics, they are very fast on large numbers of samples once the database has been loaded, and they allow for the creation and use of custom databases. Only Bracken is based on a probabilistic approach to generate the final abundance profiles. Among the protein-based classifiers, Kaiju is recommended by Ye et al. [3] to access fast and efficient classification with minimum memory requirements compared to other classifiers. The development of metagenomic analysis pipelines also includes the recently published Sunbeam based on Kraken1 and SqueezeMeta, which represents a co-assembly procedure without filtering [4,5]. Taxonomic classifiers are still burdened by high numbers of false-positive calls at low abundance. Consequently, it is essential to provide a quality review process of metagenomics data in order to validate metagenomic pathogen detection results in clinical laboratories. In a diagnosis setting, clinical microbiologists are usually very much involved in the different analytical steps such as nucleic acid extraction and sequencing with standard operating procedures and quality controls. Nevertheless, they also must be involved in the post-analytical phases of the process, i.e., the validation of all analytical data, and should build close relationships with bioinformaticians. The ultimate goal is to provide the whole workflow to accreditation.

Here, we propose a methodology for taxon identification and abundance assessment of shotgun sequencing data of microbes that are well fitted for clinical setup and routine use in diagnosis. It uses classifiers highlighted in the paper of Ye et al. [3] and accounts for limited computing resources, allowing the pipeline to be used on a routine basis in fast-decision-making processes linked to diagnostic analyses.

## 2. Materials and Methods

### 2.1. Dataset and Quality of Reads

We used the simBA525 data set tested by Ye et al. [3]. This dataset contains reads randomly chosen from 525 bacterial/archaeal species. It is composed of short synthetic reads generated using ART with default settings [6].

Before filtering and gene mapping, a robust quality review process included the k-mer analysis of raw metagenome sequence reads. We used the k-mer analysis method introduced by Onate et al. [7]. Then, we selected Trimmomatic to identify and remove low-quality sequences and contaminants, as it is recognized as particularly efficient in various studies [8,9,10].

### 2.2. Sensitivity and Specificty

To estimate a good value for a specific abundance threshold, Ye et al. used a precision-recall curve, where each point represents the precision (specificity) and recall (sensitivity) scores at a specific abundance threshold [3]. Here, we calculated a cutoff of minimal reads per species to compare Kraken and Kaiju.

### 2.3. Databases

Direct taxonomic classification is useful for quantitative community profiling and identifying organisms with close relatives in the database [11]. Among the 20 classifiers presented in the paper of Ye et al., only Bracken employs a probabilistic approach to generate the final abundance profiles [3]. Each classifier tool uses precompiled reference databases, which can differ widely. Kraken and Kaiju are distributed with precompiled reference databases similar to RefSeq (completely assembled and annotated reference genomes of archaea, bacteria, and viruses from the NCBI RefSeq database). Unlike Kraken, Kraken Uniq, and Kaiju, Kraken 2 includes additional bacterial databases such as 16S Greengenes, 16S Silva, and 16S RDP. Eukaryotic RefSeq contains only 191 of 1897 (10%) fungal genome assemblies. Kaiju was the only classifier that included a fungal database (fungal sequences from the NCBI RefSeq database) and nr_euk database (as option -s nr additionally included proteins from fungi and microbial eukaryotes). We added fungal genomes of medical interest such as *Candida parapsilosis* GCA_004026285.1, *Candida krusei* GCA_002166775, *Candida tropicalis* GCA_002864075, *Aspergillus flavus* GCF_000006275, *Aspergillus nidulans* GCF_000149205, *Scedosporium apiospermum* GCF_000732125, *Scedosporium boydii* GCA_002221725, *Trichosporon asahii* GCF_000293215, and *Geotrichum capitatum* GCA_000817185.

Furthermore, plasmid sequences and mobile genetic elements (MGEs) in the RefSeq database can lead to incorrect taxonomic classification, being a major concern. Those sequences may be shared with different bacterial species and cannot be used as a discriminatory marker for bacterial taxa. We then modified the database by separating the plasmid sequences from bacterial RefSeq genomes and re-assigned them to a single taxon for all plasmid and synthetic vector sequences, as recommended by Doster et al. [12]. Of note, an additional defect of the Kraken 2 database includes the GRCh38 assembly of the human genome [13].

### 2.4. Controls

External positive and negative controls were included in each run. A positive control was composed of one or more pathogens. Negative controls contained extraction buffer or blank transport media to identify specimen-to specimen and reagent contamination. If quantitative values are used to interpret the results, acceptable ranges need to be established during validation. The L2 distance was defined by Ye et al. as the distance between the species abundance profile of a positive control compared with the true composition [3].

## 3. Results

**Figure 1:**
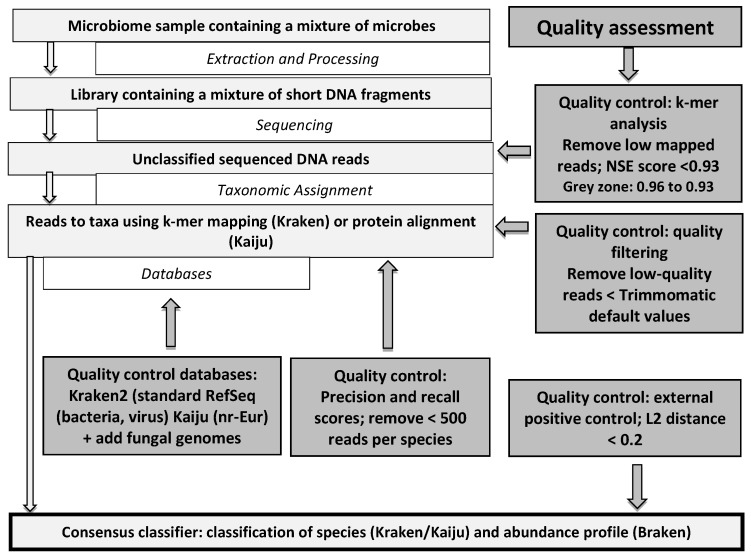
Processing steps and quality assessment for metagenomics data.

We present the key metrics for quality assessment of Kraken/Bracken and Kaiju that have a major impact on the robustness of biological and clinical conclusions drawn from metagenomic studies as a practical algorithm in Figure 1. The processing steps are as follows.

### 3.1. Quality Review of Reads

The abundance of all overlapping k-mers (with k = 4) was first computed for the set of reads. Then, the distribution of the occurrence of all 256 k-mers was evaluated using the normalized Shannon entropy (NSE), giving a score between 0 and 1. The NSE for the simBA525 data set tested by Ye et al. was 0.989746.

### 3.2. Quality Review of Sequences/Quality Filtering

Trimmomatic includes a variety of processing steps for read trimming and filtering, but the main algorithmic innovations were related to the identification of adapter sequences and quality filtering. We used the Trimmomatic default values.

### 3.3. Precision and Recall Scores across All Abundance Thresholds

**Figure 2:**
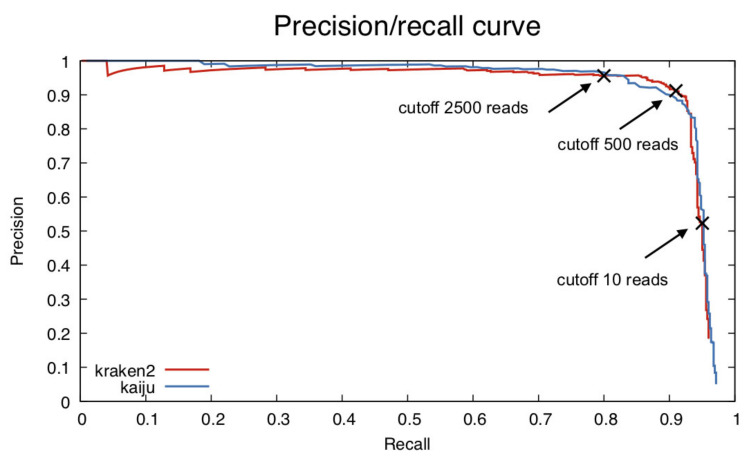
Precision/recall curves for the classifiers Kraken 2 and Kaiju with the simBA525 dataset according to Ye et al. in 2019 [3]. Each point in the curves represents the precision and recall score for a specific read abundance threshold, calculated on a simulated dataset. We observed a sharp decrease in precision when the threshold was below 500 reads per species, indicating many false-positive species with low abundance. The figure also shows the cutoff values for recall of 0.8 and 0.95 at 2500 and 10 reads, respectively.

To estimate a good value for a specific abundance threshold, we computed the precision/recall values for all possible thresholds and generated a precision/recall curve from this data (Figure 2). We observed that when decreasing the threshold, precision first decreased slowly and then dropped sharply below 500 reads per species. Therefore, we recommend a cutoff of 500 reads minimum per species.

### 3.4. Databases

Kraken 2 allowed the use of both a standard database and custom databases. The standard Kraken 2 database contained NCBI taxonomic information, as well as the complete bacterial, archaeal, and viral genome sequences in RefSeq, the human genome, and a collection of known vectors (UniVec_Core). Other genomes were also added, but such genomes have to meet certain requirements, i.e., sequences must be in FASTA format (multi-FASTA is allowed). When genomes met these requirements, each sequence was added to the database’s genomic library using the –add-to-library switch. In combination with Kraken 2, Kaiju was cleverly complete with fungal sequences from the NCBI RefSeq database by including additional proteins from fungi and microbial eukaryotes as option-s nr.

### 3.5. External Controls

An L2 distance of <0.2 with the classifier Kraken2/Bracken/Kaiju proved efficient.

### 3.6. Consensus Classifier

To summarize, the quality assessment must include an NSE > 0.96, a standard Trimmomatic quality of filtering, a minimum of 500 reads, databases from Kraken2, Bracken, Kaiju, specific fungal genomes, and an L2 distance < 0.2 for the external positive control (Figure 1).

## 4. Discussion

Deep sequencing or next-generation sequencing is now the standard to reconstruct microbial communities, including non-cultural microorganisms, and these wide datasets of taxonomic and functional diversity need robust and qualitative bioinformatic analysis [14].

### 4.1. Quality Review of Reads

As previously described, Onate et al. showed that the normalized Shannon entropy (NSE) with a score between 0 and 1 is a good indicator of the diversity and quality of the metagenomic sample. We thus propose to use the following values to evaluate dataset quality prior to more extensive bioinformatics analysis: an NSE > 0.96 denotes a good quality dataset; an NSE < 0.93 denotes a low-quality dataset and an NSE in the range of (0.93–0.96) is considered as inconclusive.

### 4.2. Quality Review of Sequences/Quality Filtering

As mentioned earlier, Trimmomatic was shown to produce output that outperforms the output produced by other tools such as Cutadapt in all scenarios tested [8,9,10]. The processing steps determine the quality of reads, divide that value by the read length, check whether the threshold is reached or not, and eliminate or retain the read. (trimmomatic version 0.40).

### 4.3. Precision and Recall Scores across All Abundance Thresholds

One of the biggest performance challenges for many classifiers is that they often report large numbers of low-abundance false positives, lowering the accuracy. As recommended by Ye et al. [3], we computed the precision/recall values to find a cutoff of 500 reads minimum per species.

### 4.4. Databases

The rapid growth in the number of reference databases represents a fundamental challenge for the clinical interpretation of the results. This emphasizes the importance of selecting databases and appropriate methods for interpreting results. The most popular reference databases are RefSeq complete genomes (RefSeq CG) for microbial species as well as the BLAST nt and nr databases for high-quality nucleotide and protein sequences, respectively (50 and 200 million sequences). Other databases include SILVA for 16S rRNA, with 2 million sequences, and GenBank, with a large number of genomes and lower quality control standards. The RefSeq database contained all the viral genomes of medical interest listed by the Society of French Virology (*Traité de virologie médicale*, Thomas Mourez, Sonia Burrel, David Boutolleau et al. 2e éd. Paris: Société française de microbiologie; Société française de virologie, 2019). Current resources for fungal identification were added in order to improve the database. For all classifiers, it was crucial to add fungal genomes of medical interest, such as *Candida parapsilosis*, *Candida krusei*, *Candida tropicalis*, *Aspergillus flavus*, *Aspergillus nidulans*, *Scedosporium apiospermum*, *Scedosporium boydii*, *Trichosporon asahii*, and *Geotrichum capitatum*. The human genome was included in the default databases of Kaiju, Kraken, KrakenUniq, and Kraken 2 (GRCh38). Reads mapping of the human genome were removed after Kraken 2 analysis for accurate quantification of microbial species.

### 4.5. External Controls

External positive and negative controls are essential for the accreditation process and were included in each run. As proposed by Ye et al. [3], an L2 distance defined as the distance between the species abundance profile of the positive control compared with the true composition was used and an L2 distance of <0.2 was considered as a good value.

### 4.6. Consensus Classifier

In this work, our strategy was to propose a complete pipeline of different classifiers in accordance with Ye et al. but dedicated to clinical microbiology in a routine setting [3]. Our experiments showed that Kraken 2 is generally a more precise classifier than Kaiju (Figure 1). However, the Kaiju protein database was more complete and allowed more reads to be classified. Therefore, we built an ensemble classifier as follows: each read was classified with Kraken 2 results, and as a fallback, reads not classified with Kraken 2 were classified using results from Kaiju (Figure 1). Sunbeam, by comparison, also provides an extensible pipeline for analyzing metagenomic sequencing data is based on Kraken 1, which demands much more computational resources and only refers to databases built for Kraken v1 [4]. SqueezeMeta is also an interesting metagenomic analysis pipeline with the characteristics of a co-assembly procedure without filtering but allows detecting only abundant species [5].

### 4.7. Potential Limitations

The aim of this work was to describe optimal quality procedures and filtering steps for a taxonomic classifier pipeline to be used on a routine basis for metagenomic analysis of clinical data, taking into account both accuracy and cost constraints (compute resource, process time). This optimized pipeline resulted in a combination of classifiers previously described by Ye et al. [3] improved with specific quality steps and thresholds to be applied.

While the scope of this work was not to present a new classifier for all types of datasets, the presented optimized pipeline is a good candidate for additional analyses on different groups of microorganisms and interlaboratory collaborations to further describe its sensitivity and specificity.

## 5. Conclusions

Genome reconstruction of the microbial population is supported by the classification of individual reads or contigs and the profile of microorganism proportions. With the increasing demand for metagenomic analysis of microbiota in medical microbiology, it is crucial to develop tools for rapid and efficient decision-making. This will eventually lead to a faster turn-around time, improved analytical quality, including sample quality metrics, and a significant cost reduction. Improved quality assessment has a major impact on the robustness of biological and clinical conclusions drawn from metagenomic studies. New developments are ongoing in this fascinating topic of a computational framework for taxonomic classification. As an example, a deep learning-based computational framework for taxonomic classification called DeepMicrobes avoiding the lack of a well-curated taxonomic tree is described [15]. Using this new tool, the authors reported potential novel signatures in inflammatory bowel diseases [16]. Definitely, machine learning approaches for taxonomic classification of metagenomics data will ensure quality improvement of pipelines for a better understanding of factors affecting microbial communities and functions [16,17]. Then, the following step will be to integrate metagenomics data in an integrative systems medicine approach also combining metabolomics and transcriptomics to decipher the pathophysiology of many systemic diseases [18].

## Data Availability

The datasets used an analyzed during this study are available from the corresponding author on reasonable request.
